# Cognitive impairment and prefrontal TGF-β1 elevation in a rat model of fatigue

**DOI:** 10.3389/fpsyt.2026.1841951

**Published:** 2026-06-19

**Authors:** Yingru Wu, Xuan Wen, Zeman Fang, Jinling Zhang, Handi Zhang

**Affiliations:** 1 Shantou University Mental Health Center, Shantou, China; 2 Shantou University Medical College – Faculty of Medicine of University of Manitoba Joint Laboratory of Biological Psychiatry, Shantou University Mental Health Center, Shantou, China

**Keywords:** chronic fatigue syndrome, cytokines, mental fatigue, TGF-β1, working memory

## Abstract

**Introduction:**

Chronic fatigue syndrome (CFS) is a complex disorder of unknown etiology, characterized by persistent fatigue unrelieved by rest and accompanied by cognitive dysfunction. While dysregulated cytokines are implicated in CFS pathogenesis, the role of anti-inflammatory transforming growth factor β1 (TGF-β1) remains poorly defined.

**Methods:**

This study investigated central and peripheral TGF-β1 dysregulation and cognitive function in a rat model of fatigue induced by 10-day repetitive sleep deprivation with intermittent rest. Rats were randomly divided into the control group and the fatigue group. Behavioral tests including open field test and Y-maze test were performed after the end of fatigue-loading procedure. Peripheral and central TGF-β1 levels were detected.

**Results:**

Rats in the fatigue group exhibited unchanged daytime short-term locomotor activity but significantly increased anxiety-like behavior in the open field test. In the Y-maze test, the fatigue group showed markedly reduced spontaneous alternation rates compared to controls. Furthermore, prefrontal cortical TGF-β1 levels were elevated in fatigued rats, whereas neither peripheral nor striatal TGF-β1 differed between groups.

**Conclusion:**

These findings demonstrate that the 10-day repetitive sleep deprivation with intermittent rest fatigue model induces cognitive impairment and increased anxiety-like behavior, with selective prefrontal TGF-β1 upregulation. The concomitant elevation of prefrontal TGF-β1 and cognitive deficits suggests a potential role for central TGF-β1 signaling in the pathophysiology of mental fatigue, although the precise nature of this relationship remains to be elucidated.

## Introduction

Chronic fatigue syndrome (CFS) is a complex disorder with an unknown etiology, characterized by persistent fatigue lasting for more than six months that is unrelieved by rest, accompanied by comorbid symptoms including pain, cognitive and emotional impairment (1). The global prevalence of CFS is estimated to be between 17 and 24 million individuals, representing approximately 1% of the population (2). Prolonged fatigue can lead to a notable decline in social functioning, impacting the quality of life and work efficiency (3). There is a pressing need to investigate the underlying psychophysiology mechanisms of CFS.

Although the precise etiology of CFS is unclear, accumulating evidence implicates immune dysregulation and cytokine imbalance in its pathogenesis (4). Clinical studies consistently report aberrant cytokine profiles in CFS patients (5), with elevated peripheral transforming growth factor-β1 (TGF-β1) emerging as a particular potential biomarker. Systematic reviews confirm that among multiple cytokines altered in CFS, TGF-β1 shows the most consistent elevation in peripheral blood (6, 7). Preclinical evidence further supports its mechanistic role. In rodent models, Poly I:C-induced fatigue is associated with reduced spontaneous activity and elevated TGF-β1 levels in the cerebrospinal fluid in rats (8); correspondingly, intracerebroventricular administration of TGF-β1 recapitulates fatigue-like behaviors in mice (9). Collectively, these findings suggest TGF-β1 may be critically involved in CFS pathophysiology.

TGF-β1 is a pleiotropic cytokine with predominantly anti-inflammatory functions (10). While peripherally it regulates T-cell differentiation (10), it is also widely expressed in the central nervous system by neurons and glia (11, 12). Beyond its roles in neurodevelopment (13–15), TGF-β1 modulates neurotransmitter systems like dopamine (16), serotonin (17), glutamate (8), neurorepair (18), angiogenesis (19), synaptic plasticity (20), and cognitive processes (21).

Despite these advances, the questions whether peripheral TGF-β1 elevation reliably reflects central dysregulation in CFS, and how TGF-β1 dynamics correlate with disease progression and its contribution to CFS-associated cognitive deficits remain unresolved. In the present study, we aimed to investigate the cognitive and emotional functions and central and peripheral TGF-β1 alterations in an established rat model of chronic fatigue (22, 23).

## Materials and methods

### Animals

Twenty-two healthy male Sprague-Dawley rats (aged 4–5 weeks; initial body weight 200–230 g) were obtained from Vital River Laboratories (Beijing, China). All animals were specific pathogen-free and maintained under standard laboratory conditions with temperature 23 ± 1 °C, 12-h light/dark cycle (lights on at 07:00) and ad libitum access to standard rodent chow and tap water. After a 7-day acclimation period, rats were randomly assigned to experimental groups. To minimize stress during behavioral testing, animals were gently handled twice daily for 2 min per session throughout the acclimation phase. All experimental procedures were approved by the Ethics Committee of Shantou University Medical College on animal care and use.

### Fatigue model and experimental procedures

The rat fatigue model was established based on previous reports (22, 23). Briefly, rats acclimatized for one week and then were randomly divided into control and fatigue groups. Rats in the fatigue group were exposed to fatigue-loading (repetitive sleep deprivation with intermittent rest) for 10 days. During the daytime (9:00 to 19:00), rats in the fatigue group were individually housed in plastic cages of 50 cm × 20 cm × 19 cm filled with water (23 ± 1°C) to a depth of 2.2 cm. The rats were allowed to rest for 5 mins twice per hour in a standard cage at variable interval. After each time of rest, rats were transferred back to the previous water-filled cage. Each rat in the fatigue group was given a total of 20 breaks per day. The water was changed twice daily, and the bedding was changed once daily. At night (19:00 to 9:00), rats in the fatigue group were placed in a sleep-deprivation device (single-platform method) to deprive their rapid eye movement sleep for the whole night as described before (24). The sleep-deprivation device consisted of a small platform with a diameter of 6.3 cm, and a height of 8 cm positioned 1 cm above water in enclosed tanks. The rats in the control group were housed individually in standard cages without any fatigue-loading except for daily handling by the experimenter.

**Figure 1:**
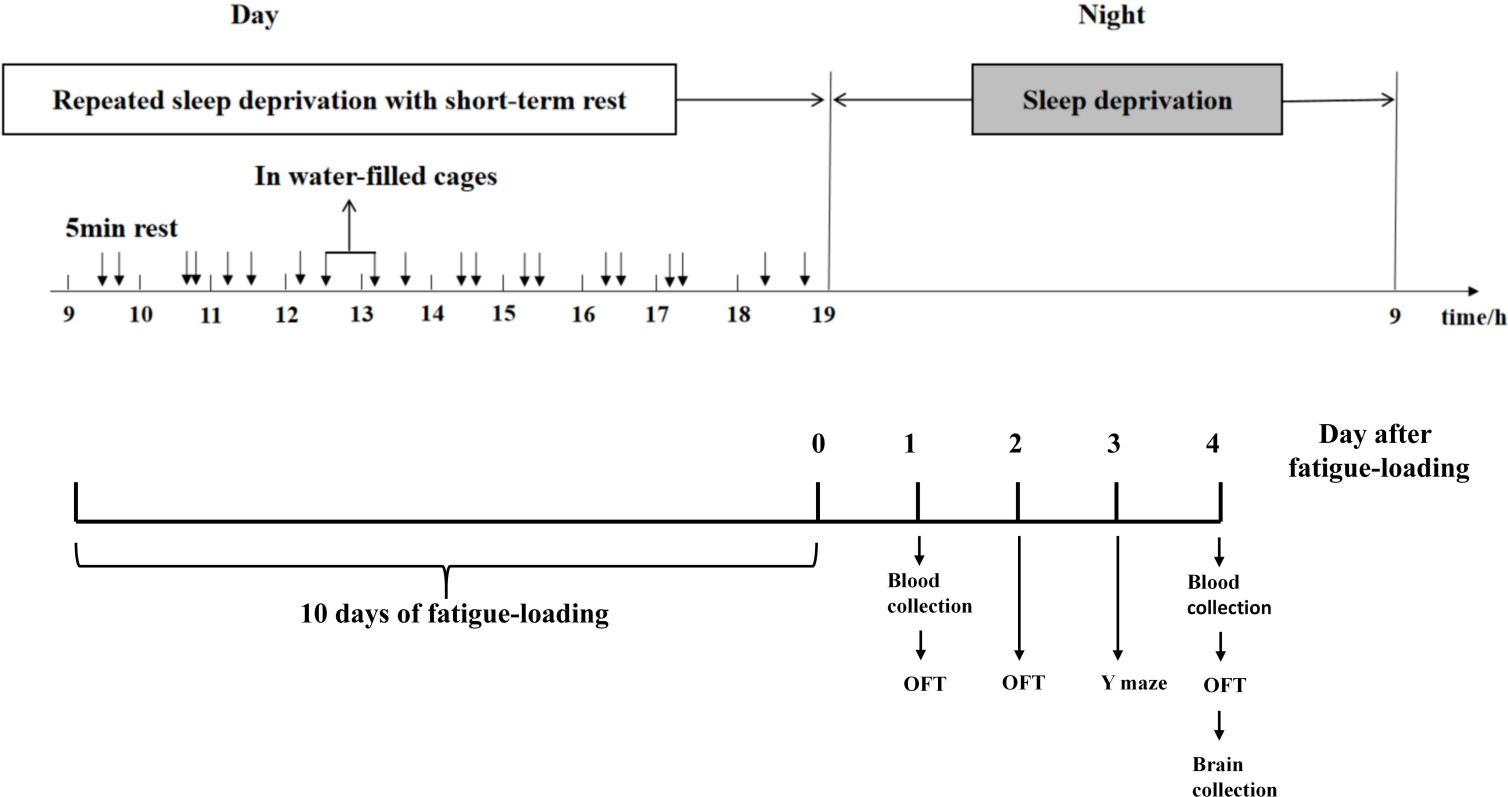
The experimental procedure. OFT, open field test.

The experimental procedure is shown in **Figure 1**. Rats in both groups were subjected to behavioral tests, including open field test (OFT) and Y-maze test after the end of fatigue-loading. OFT were performed on day 1, 2 and 4 post-fatigue loading to dynamically evaluate the daytime short-term locomotor activity, which was used as an index of physical fatigue. Y-maze test was performed on day 3 post-fatigue loading to evaluate the spatial working memory, which was used as an index of mental fatigue. Tail vein blood was collected on day 1 and 4 post-fatigue loading for measuring peripheral TGF-β1 levels. All the rats were sacrificed under deep anesthesia, and their brains were processed for Western blot on day 4 post-fatigue loading.

### OFT

Locomotor activity and anxiety-like behavior were assessed using the OFT on days 1, 2, and 4 post-fatigue loading. Rats were habituated to the testing room for 60 min before the test. Each rat was placed in the center of a square arena (100 × 100 × 40 cm) and allowed to explore freely for 5 min. Sessions were recorded by an overhead camera and analyzed using EthoVision XT 9.0 (Noldus, Netherlands). The arena was cleaned with 70% ethanol between trials to eliminate odor cues.

### Y-maze test

Spatial working memory was evaluated by spontaneous alternation in the Y-maze test on day 3 post-fatigue loading. The maze consisted of three identical arms (30 cm long × 20 cm high × 8 cm wide) angled at 120° with a central junction. Rats were placed at the center junction facing a randomly selected arm and allowed 8 min of free exploration. The alternation sequence was recorded when a rat entered three different arms consecutively (e.g., A→B→C). Spontaneous alternation rate (%) was calculated as: number of correct arm entries/(total number of arm entries– 2) × 100%. Trials were video-recorded and analyzed using EthoVision XT 9.0. The maze was cleaned with 70% ethanol after each trial.

### Peripheral blood TGF-β1 measurement

Rats were anesthetized with isoflurane, and the tail tips of rats were quickly clipped by 1–2 mm, then about 1 ml of blood were collected using blood collection tubes without anticoagulant. The wound was pressed with a cotton ball for 1–2 min. Whole blood was placed at room temperature for natural coagulation for 30 min, centrifuged at 3000 rpm at 4 °C for 15 min, and the top yellow serum was taken and frozen at -80 °C. The levels of TGF-β1 in serum were detected using an Enzyme-Linked Immunosorbent Assay (ELISA) kit according to the manufacturer’s protocols (Cat #: EK0514, BOSTER, Wuhan, China).

### Preparation of rat brains

Rats were anesthetized with isoflurane, rapidly decapitated, and brains were immediately removed. The prefrontal cortex (PFC) and striatum were then quickly dissected on a chilled plate, flash-frozen on dry ice, and stored at -80 °C until further use.

### Western blotting and densitometric analysis

The prefrontal cortex and striatum samples were homogenized in ice-bathed radioimmunoprecipitation assay (RIPA) lysis buffer (Cat #: P0013B, Beyotime, Shanghai, China) with freshly added protease inhibitor phenylmethanesulfonylfluoride (PMSF; Cat #: ST506, Beyotime, Shanghai, China). Then the suspension was centrifuged at 12,000 rpm for 10 min at 4 °C. The total protein concentration was determined by bicinchoninic acid (BCA) assay (P0009, Beyotime, Shanghai, China). Total proteins (45 µg) were loaded onto a 12.5% sodium dodecyl sulfate polyacrylamide gel and subjected to electrophoresis at 70 V and 120 V until the bromophenol blue reached the bottom of the gel. The proteins were then transferred to a nitrocellulose membrane at 72 V for 65 min in an ice-cooled transfer apparatus. The membrane was blocked in the blocking buffer (5% skim milk in 1× tris-buffered saline with Tween-20 (TBST)) for 2 h at room temperature and then incubated for 1.5 h at room temperature in primary antibody against TGF-β1 (1:1,000; Cat #: ab179695; Abcam, United Kingdom) diluted in the blocking buffer. The membranes were then washed with 1× TBST three times (10 min each), followed by incubation in the blocking buffer containing horseradish peroxidase (HRP)-coupled secondary antibody (1:5,000, Beyotime, Shanghai, China) for 1 h at room temperature. After three rinses in 1× TBST (10 min each), immunoreactive bands were developed using an excellent chemiluminescent (ECL) substrate detection kit (Cat #: P0018AS; Beyotime, Shanghai, China) and imaged on a Bio-Rad ChemiDoc XRS. Band intensities were quantified using ImageJ software, and TGF-β1 protein levels were normalized to GAPDH. To confirm equal amounts of loading samples, glyceraldehyde-3-phosphate dehydrogenase (GADPH) was detected with a primary antibody (1:1,000; Cat #: AG019; Beyotime, Shanghai, China) through the same procedures as described above.

### Statistical analysis

The data were presented as mean ± standard deviation (SD) and tested for normality using the Shapiro-Wilk test. OFT data were analyzed by two-way repeated-measures ANOVA with time (three levels) as the within-subject factor and group (two levels) as the between-subject factor. The Greenhouse-Geisser correction was used when sphericity was violated. When the interaction was not significant, *post-hoc* comparisons between time points were performed on the pooled sample using the Bonferroni correction. No *post-hoc* test was applied for the group factor because it consisted of only two levels. The original OFT data are provided in **Supplementary Data S1**. Y maze spontaneous alternation rates and TGF β1 levels were compared between groups using independent samples t tests. A *post-hoc* power analysis for the Western blot data is provided in the **Supplementary Materials**. Differences were considered statistically significant at *P* < 0.05. All statistical analyses were performed using SPSS version 23.0 for windows.

## Results

### Fatigue-loading induced altered anxiety-like behavior in the OFT

OFT was conducted on the morning of days 1, 2, and 4 post-fatigue loading to dynamically assess spontaneous locomotor activity and anxiety-like behavior.

**Figure 2:**
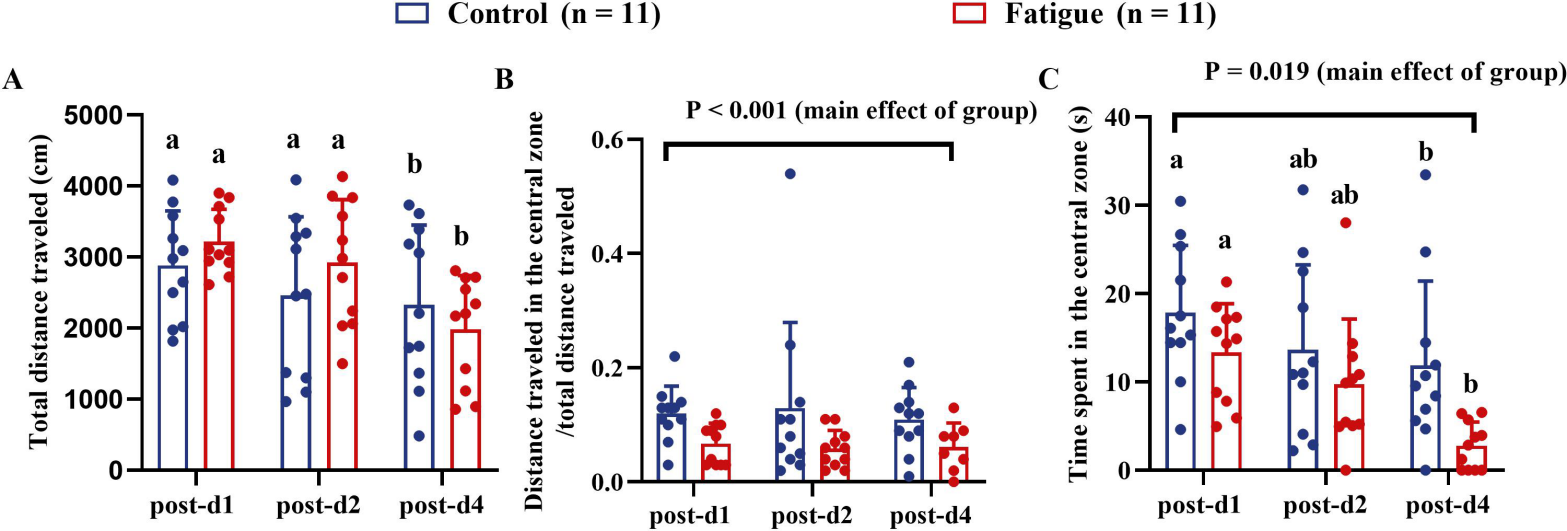
Effects of fatigue-loading on locomotor activity and anxiety-like behavior in rats. The OFT was performed on post-d1, d2, and d4. Data are mean ± SD (n = 11/group), analyzed by two-way repeated-measures ANOVA with Bonferroni *post-hoc* correction. **(A)** Total distance traveled. Main effect of time: P < 0.001. No significant group effect or interaction. Different lowercase letters denote significant differences between time points after Bonferroni correction (a vs. b, P < 0.05). **(B)** Ratio of central zone distance to total distance. Main effect of group: P < 0.001, partial η² = 0.460. The fatigue group showed a lower ratio than controls overall (mean difference = 0.063, 95% CI 0.031–0.094). No significant time effect or interaction. **(C)** Duration in the central zone. Significant main effects of time (P < 0.001) and group (P = 0.019), with no significant interaction. Different lowercase letters denote significant time differences after Bonferroni correction (a vs. b, P < 0.05). The fatigue group spent less time in the central zone than controls overall (mean difference = 5.85, 95% CI 1.05–10.64). OFT, open field test; post-d1, post fatigue-loading day 1; post-d2, post fatigue-loading day 2; post-d4, post fatigue-loading day 4; SD, standard deviation.

Analysis of total distance traveled revealed a significant main effect of time [F (2, 40) = 9.441, P < 0.001, partial η² = 0.321], while the main effect of group [F (1, 20) = 0.278, P = 0.604] and the time × group interaction [F (2, 40) = 2.209, P = 0.123] were not significant. Groups were pooled for *post hoc* comparisons: day 1 and day 2 each showed greater total distance than day 4 (P = 0.002 and P = 0.013, respectively), with no difference between day 1 and day 2 (P = 0.389) (**Figure 2A**). For the ratio of central zone distance to total distance, the main effect of group was significant [F (1, 20) = 17.028, P < 0.001, partial η² = 0.460], with the fatigue group showing a lower ratio than controls (mean difference = 0.063, 95% CI 0.031–0.094, P = 0.001). Neither the main effect of time [F (1.19, 23.70) = 0.336, P = 0.604] nor the time × group interaction [F (1.19, 23.70) = 0.076, P = 0.826] was significant (**Figure 2B**). Furthermore, analysis of duration in the central zone revealed significant main effects of time [F (2, 40) = 9.514, P < 0.001, partial η² = 0.322] and group [F (1, 20) = 6.472, P = 0.019, partial η² = 0.244], while the time × group interaction was not significant [F (2, 40) = 1.100, P = 0.343]. Groups were pooled for time-point comparisons: day 1 showed a longer duration than day 4 (P = 0.001), while day 1 vs. day 2 (P = 0.157) and day 2 vs. day 4 (P = 0.075) did not differ significantly. The fatigue group spent less time in the central zone than controls overall (mean difference = 5.85, 95% CI 1.05–10.64, P = 0.019) (**Figure 2C**).

### Fatigue-loading impairs working memory in Y-maze test

**Figure 3:**
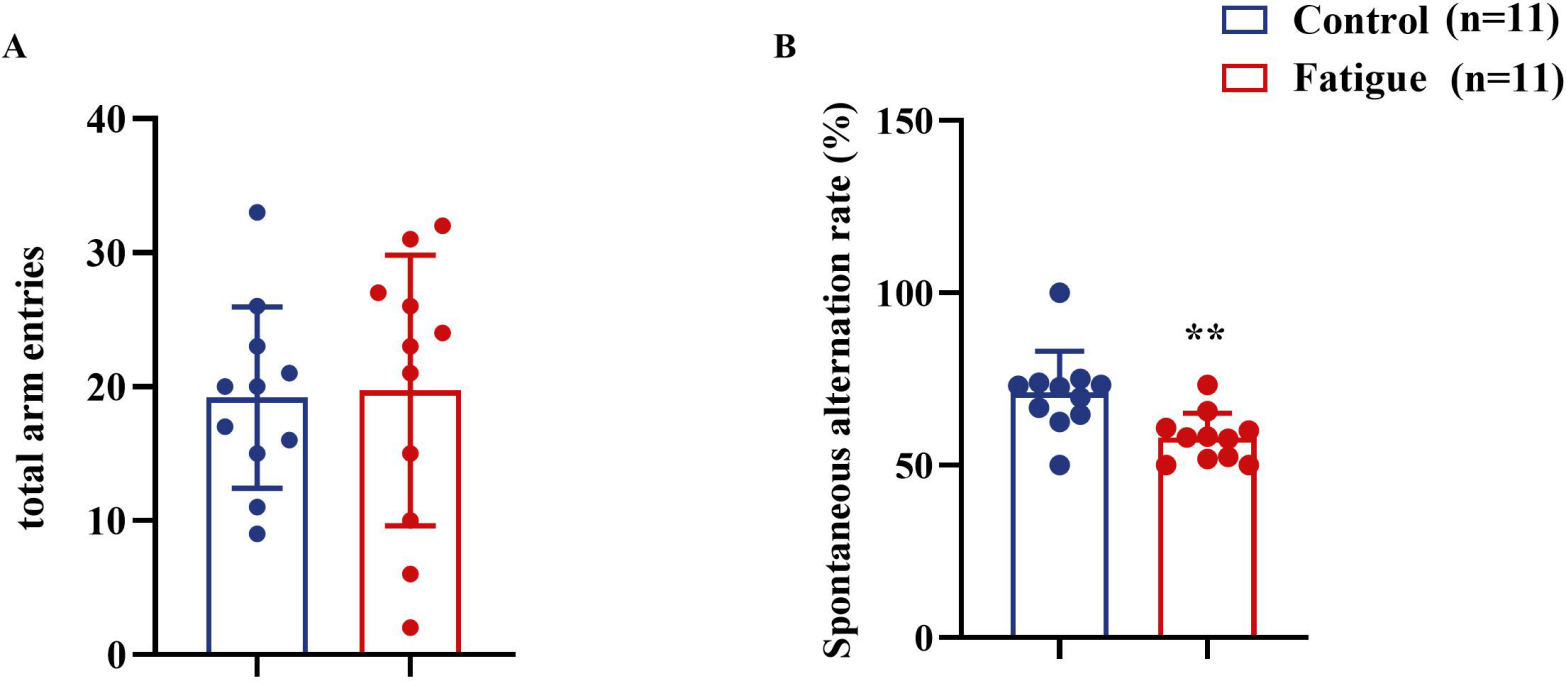
Effects of fatigue-loading on spatial working memory in rats. The Y-maze test was performed on post fatigue-loading day 3. The bar graph showed the quantitative data of total arm entries **(A)** and spontaneous alternation rate **(B)**. Data are presented as mean ± SD (N = 11 for each group). *P < 0.05 compared with the control group. SD, standard deviation.

Working memory was assessed using the Y-maze test on day 3 post-fatigue loading. Rats in the fatigue group showed a significantly lower spontaneous alternation rate compared to the control group (**Figure 3B**), whereas no significant difference was observed in the total number of arm entries between the two groups (**Figure 3A**).

### Central but not peripheral TGF-β1 elevation in fatigued rats

**Figure 4:**
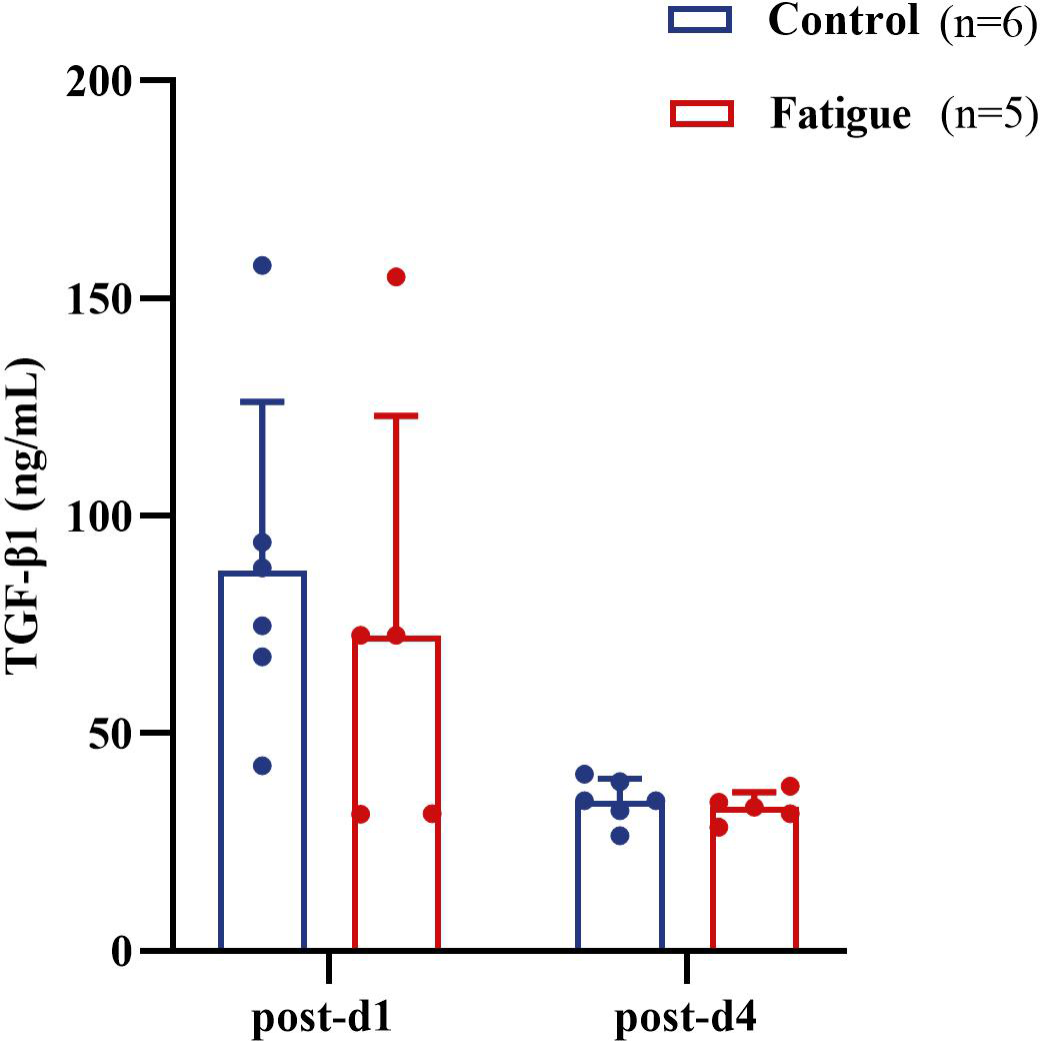
Effects of fatigue-loading on the TGF-β1 levels in serum. The bar graph showed the quantitative data of the serum levels of TGF-β1 measured using ELISA on days 1 and 4 post fatigue-loading. Data are presented as mean ± SD (N = 6 for the control, N = 5 for the fatigue group). *P < 0.05 compared to the control group. TGF-β1, transforming growth factor-β1; SD, standard deviation.

**Figure 5:**
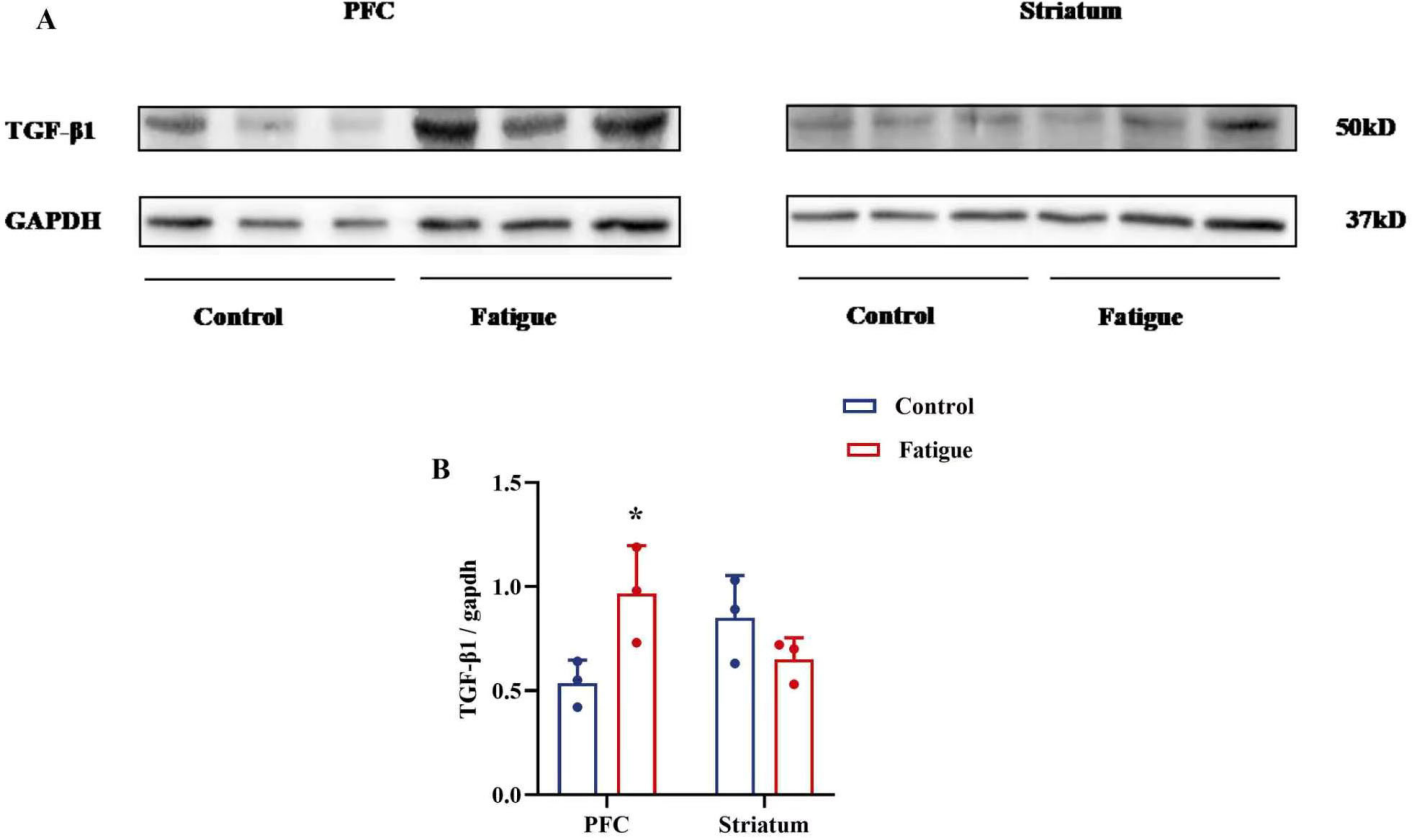
Effects of fatigue-loading on the TGF-β1 levels in PFC and striatum. TGF-β1 levels were evaluated on day 4 post fatigue-loading. **(A)** The left graph showed a representative Western blot image of TGF-β1 and GAPDH immunoreactive bands from PFC. The right graph showed a representative Western blot image of TGF-β1 and GAPDH immunoreactive bands from striatum. **(B)** The bar graph showed the quantitative data of the relative amount of TGF-β1 in PFC and striatum. Data are presented as mean ± SD (N = 3 for each group). *P < 0.05 compared with the control group. PFC, prefrontal cortex; TGF-β1, transforming growth factor-β1; SD, standard deviation.

No significant difference in peripheral blood TGF-β1 levels was observed between the fatigue and control on days 1 and 4 post-fatigue loading (**Figure 4**). However, on day 4 post-fatigue loading, TGF-β1 levels in the PFC were significantly elevated in the fatigue group compared to controls, while TGF-β1 expression in the striatum remained comparable between the two groups (**Figure 5**) (see **Supplementary Figure 1**).

## Discussion

This study establishes that a 10-day repetitive sleep deprivation with intermittent rest-induced fatigue model causes spatial working memory deficits and increased anxiety-like behavior in rats, concomitant with selective elevation of TGF-β1 in the PFC. Interestingly, these changes occurred without peripheral TGF-β1 alterations or striatal involvement, suggesting a possible role for PFC-specific neuroinflammation in fatigue-related cognitive pathology.

Our study provides the first evidence that rats subjected to repetitive sleep deprivation with intermittent rest exhibit significant working memory impairment in the Y-maze test, which is consistent with well-documented working memory impairments in CFS patients (25–27). Several studies have established a strong correlation between mental fatigue and working memory performance (28, 29). These findings suggest that the working memory impairment observed in our fatigue model effectively mirrors the cognitive aspects of mental fatigue.

Our data showed that the short-term daytime spontaneous activity in the OFT was comparable between the fatigue and control groups during post-loading days 1-4. Previous studies employing this model reported decreased swimming performance in the weight-loaded swimming test immediately after the end of fatigue-loading, with recovery observed by the following day (22). These observations suggest that repetitive sleep deprivation with intermittent rest can induce short-term physical fatigue with recovery occurring within 24 hours post-loading. Previous studies on this model also revealed that nocturnal continuous spontaneous activity was significantly decreased within 48 hours after the end of fatigue-loading, and then recovered gradually (22, 23). The differential effects observed across behavioral paradigms may be attributed to their distinct physiological underpinnings. Nocturnal continuous spontaneous activity is considered to reflect long-term endurance capacity and may be more closely associated with mental fatigue (30). This endurance impairment appears to be mediated by central mechanisms involving increased perceived effort and diminished motivation (31). Weight-loaded swimming and daytime short-term activity primarily assess anaerobic performance and acute motor reactivity, which appears relatively resistant to mental fatigue (32). The intermittent deprivation protocol adopted here was chosen over continuous sleep deprivation because prolonged continuous deprivation is associated with high mortality in rats, while intermittent paradigms produce more persistent physiological and behavioral consequences (22, 23, 47).

Notably, in the OFT, the fatigue group exhibited significantly reduced central zone exploration compared with controls, as evidenced by both a lower ratio of central/total distance moved and shorter central zone dwelling time. This pattern of reduced center exploration without locomotor impairment is classically interpreted as increased anxiety-like behavior. However, the absence of a significant time × group interaction indicates that this behavioral alteration was stable across post-loading days and did not simply reflect acute stress reactivity. Reduced center exploration together with decreased nocturnal spontaneous activity suggests a generalized suppression of exploratory drive (23). This may reflect the cognitive and affective dimensions of mental fatigue rather than a selective deficit in behavioral inhibition.

These findings collectively suggest that repetitive sleep deprivation with intermittent rest-based fatigue-loading produces significant impairment in endurance performance, which is indicative of sustained mental fatigue, with relatively short-term peripheral physical fatigue that recovers rapidly. The generalized reduction in exploratory behavior—encompassing both central zone exploration and nocturnal sustained activity—is consistent with the multifaceted behavioral phenotype of mental fatigue. However, without complementary behavioral assays such as the elevated plus maze or light-dark box, the contribution of altered anxiety levels cannot be fully excluded. We acknowledge that the dual-stressor protocol—combining sleep deprivation with water immersion—introduces multiple potential confounders, including physical exertion, psychological stress, and sleep loss. The present design cannot dissociate the relative contributions of these individual factors, and our findings should be interpreted as reflecting their combined effect.

Our study examined TGF-β1 levels in the prefrontal cortex and striatum. These regions were selected because the fatigue model used here was adapted from Li et al. (23), who originally investigated the prefrontal cortex, hippocampus, and striatum, and because both regions have been identified as core nodes in the neurocircuitry of mental fatigue (45, 46). We observed a selective increase in TGF-β1 levels in the prefrontal cortex, with no significant changes in either the striatum or peripheral blood. Whether this selectivity reflects a true region-specific process or the limited scope of regions examined remains to be determined. TGF-β1 exhibits complex, context-dependent effects in the CNS: while it demonstrates neuroprotective and anti-inflammatory properties (33, 34), excessive levels can inhibit stem cell proliferation and impair neurogenesis (35). Previous studies have shown that elevated proinflammatory cytokines in the prefrontal cortex can induce cognitive impairment (36), while TGF-β1 may help mitigate such deficits through its anti-inflammatory actions. For instance, increased TGF-β1 expression may ameliorate lipopolysaccharide-induced memory impairment (37), and TGF-β1 administration improves cognitive function in demyelination models while reducing proinflammatory factor expression (38). TGF-β1 is essential for interleukin (IL)-10-mediated suppression of microglial activation and reduction of IL-1β (39). Moreover, physiological upregulation of TGF-β1 in the healthy rat prefrontal cortex enhances memory consolidation (40). Additionally, previous studies suggest TGF-β1 may participate in both physical and mental fatigue. Lee et al. (2020) reported that adrenalectomy-induced CFS models show significantly elevated serum and CNS TGF-β1 levels accompanied by reduced muscle strength (41). Intense physical exercise can induce TGF-β accumulation in cerebrospinal fluid, contributing to central fatigue (8, 42), while direct intracerebral TGF-β1 injection induces fatigue-like behaviors in mice (9). Taken together, these findings suggest that TGF-β1 may play dual roles in fatigue pathogenesis: while potentially contributing to physical fatigue, it may also serve protective functions against mental fatigue. In our study, the selective prefrontal TGF-β1 elevation accompanied by cognitive impairment but minimal physical fatigue manifestations indicates that this model primarily induces mental fatigue associated with prefrontal dysfunction, and that the concomitant elevation of prefrontal TGF-β1 is correlated with this phenotype. While the present data cannot establish causality, we speculate that the increased TGF-β1 expression may represent an endogenous anti-inflammatory defense mechanism. Alternatively, chronic over-expression of TGF-β1 may itself contribute to cognitive dysfunction (35); the present correlative data cannot distinguish between these possibilities. Notably, while we observed no changes in peripheral TGF-β1 levels, clinical studies also reported inconsistent findings regarding circulating TGF-β1 in CFS patients (6, 7, 43, 44). The absence of peripheral changes may reflect the early, CNS-predominant phase of pathology captured by this model, the limited sensitivity of serum ELISA, or compartment-specific neuroimmune regulation (46). However, whether these factors account for the discrepancy requires further investigation. Future studies employing targeted interventions are needed to determine the causal role of central TGF-β1 changes in fatigue and cognitive manifestations.

## Conclusion

Our study demonstrates that ten days of repetitive sleep deprivation with intermittent rest-based fatigue model induces working memory deficits and increased anxiety-like behavior, accompanied by a selective elevation of TGF-β1 in the prefrontal cortex. No significant changes were detected in the striatum or peripheral blood. These correlative findings suggest that prefrontal TGF-β1 signaling may be associated with the cognitive and behavioral manifestations of mental fatigue. Causality cannot be inferred from the present data; future interventional studies are warranted to determine whether this pathway represents a viable therapeutic target for fatigue-related conditions.

## Limitations

Several limitations should be noted. The Western blot analyses were based on a small sample (n = 3/group), and the striatal null finding should be interpreted with caution. The present data are correlational, and causality cannot be inferred. Only male rats were used, which limits translational relevance to female patients given the female predominance in CFS. Tissue was collected on day 4 post-loading; whether TGF-β1 elevation represents a persistent fatigue-related change or a compensatory response remains unclear. The dual-stressor protocol cannot dissociate the individual contributions of sleep loss, physical exertion, and psychological stress. Only the prefrontal cortex and striatum were examined, and anxiety-specific behavioral assays were not included. Future studies addressing these limitations are warranted.

## Data Availability

The raw data supporting the conclusions of this article will be made available by the authors, without undue reservation.
