# Supplementary material for: Cognitive impairment and prefrontal TGF-β1 elevation in a rat model of fatigue
Source: Front Psychiatry. 2026 Jun 19;17:1841951. doi: 10.3389/fpsyt.2026.1841951 (PMC13328434; doi:10.3389/fpsyt.2026.1841951)

Supplementary Methods. Post-hoc power analysis for Western blot data

A post-hoc power analysis was conducted using G*Power (version 3.1) to evaluate the achieved statistical power of the Western blot comparisons given the small sample size (n = 3 per group). The analysis was performed for the two key brain regions examined: the prefrontal cortex (PFC) and the striatum.

Parameters: Two-tailed independent-samples t-test; $\alpha = 0.05$; allocation ratio N2/N1 = 1; sample size group 1 = 3, sample size group 2 = 3. Effect size was entered as Hedges' g, which corrects Cohen's d for small-sample bias.

Prefrontal cortex: The observed Hedges' g = 2.385. The achieved power (1-β) = 0.60. Although this power is below the conventional threshold of 0.80 due to the small sample, the very large effect size (g = 2.385) and the complete separation of individual data points between groups (see Supplementary Figure S1) support the robustness of the observed difference. The statistical significance reached (P = 0.043) despite this limited power further underscores the magnitude of the effect.

Striatum: The observed Hedges' g = 1.265. The achieved power (1-β) = 0.22, indicating that the sample was severely underpowered to detect a difference of this magnitude (see Supplementary Figure S2). Therefore, the non-significant P-value (0.204) should not be interpreted as evidence of no effect, but rather as reflecting insufficient statistical power. This limitation has been explicitly acknowledged in the Discussion.

These results indicate that the PFC TGF-β1 elevation is a robust finding, whereas the striatal null result remains inconclusive due to low statistical power. Future studies with larger sample sizes ($n \geq 6$ per group) are recommended to confirm the regional specificity of TGF-β1 alterations in this model.

Supplementary Figure S2. Post-hoc power analysis output from G*Power

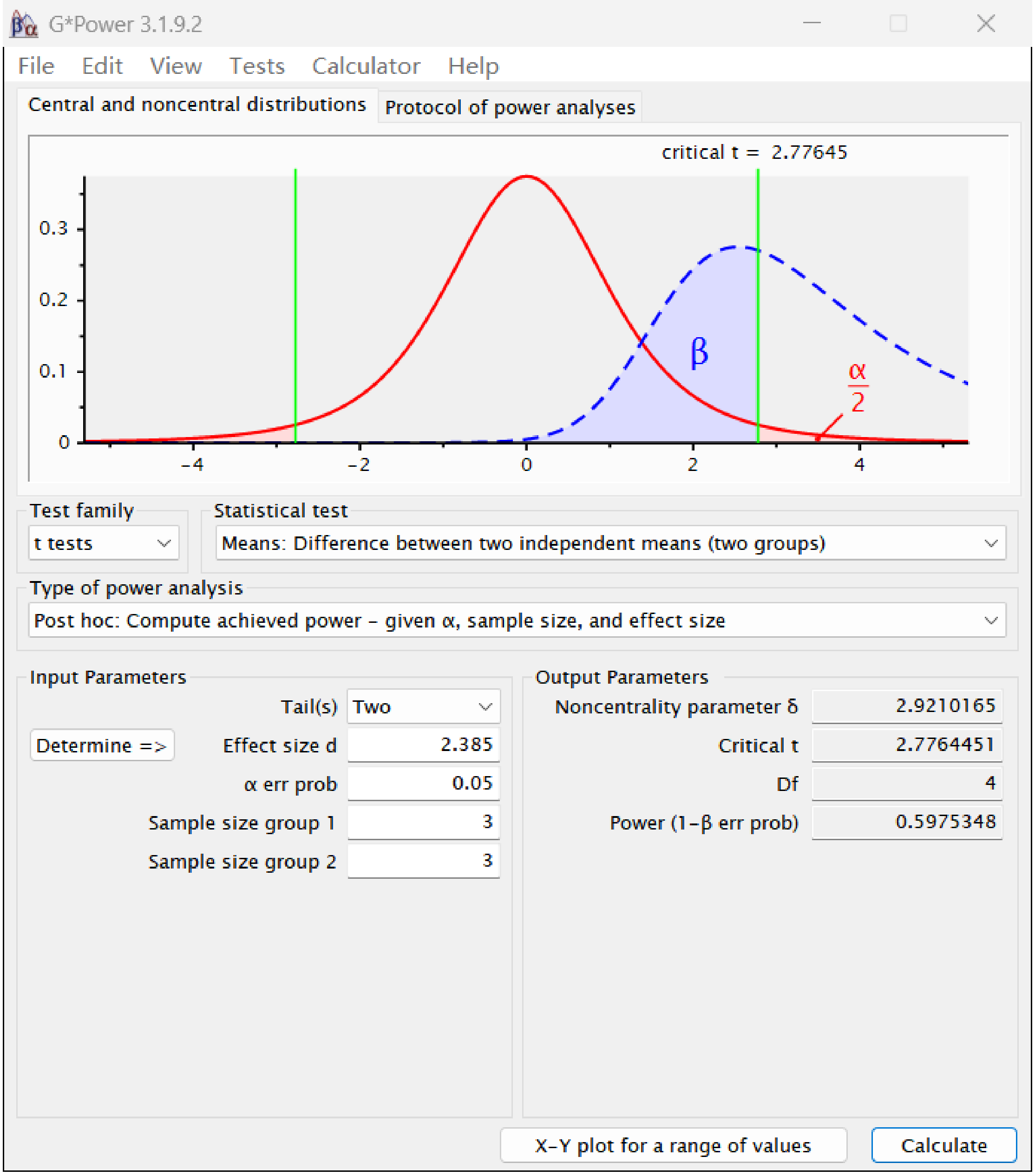

S2.1. The analysis was conducted for the prefrontal cortex TGF-β1 comparison (two-tailed independent-samples t-test, $\alpha = 0.05$, n1 = n2 = 3). Hedges' g = 2.385 was entered as the effect size. The achieved power (1-β) = **0.60**. Parameters are shown in the input panel (left), and output is displayed in the protocol panel (right).

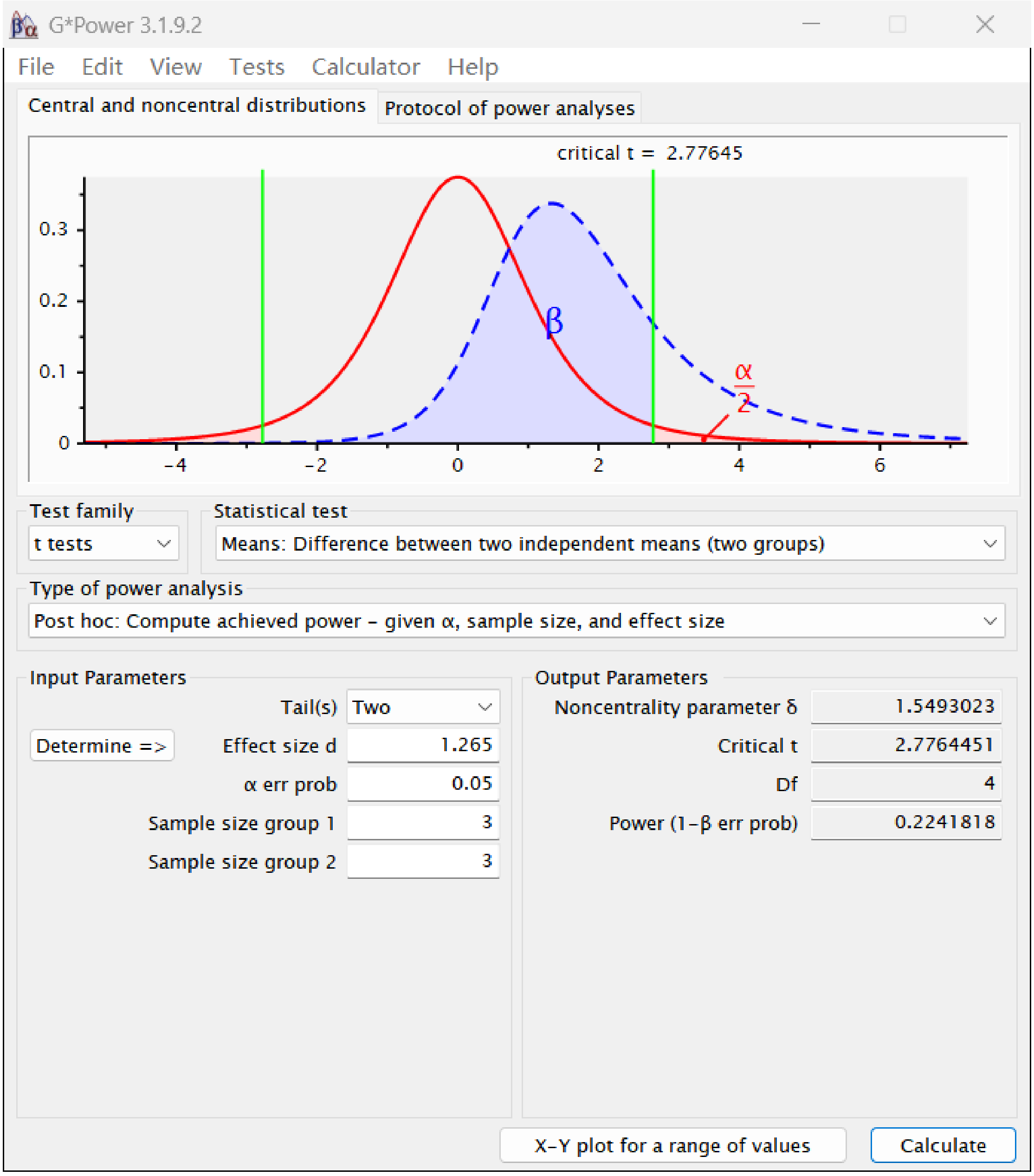

S2.2. Post-hoc power analysis output from G*Power 3.1 for striatal TGF-β1 comparison. The analysis was conducted using a two-tailed independent-samples t-test ($\alpha = 0.05$, $n1 = n2 = 3$). Hedges' g = **1.265** was entered as the effect size. The achieved power $(1\text{-}\beta)$ = **0.22**, indicating that the sample was severely underpowered to detect a difference of this magnitude. Therefore, the non-significant P-value (0.204) should not be interpreted as evidence of no effect.

Supplementary Figure S3. Individual data points for Western blot analyses. Each dot represents one animal (n = 3 per group).

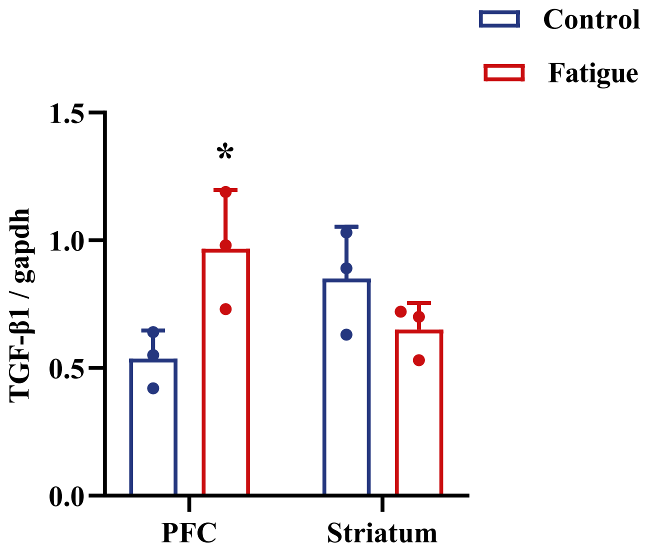

Supplement: Supplementary file 1 [file Supplementaryfile1.zip › Supplementary Materials.PDF]
