# Supplementary figures and images for: Cognitive impairment and prefrontal TGF-β1 elevation in a rat model of fatigue
Source: Front Psychiatry. 2026 Jun 19;17:1841951. doi: 10.3389/fpsyt.2026.1841951 (PMC13328434; doi:10.3389/fpsyt.2026.1841951)

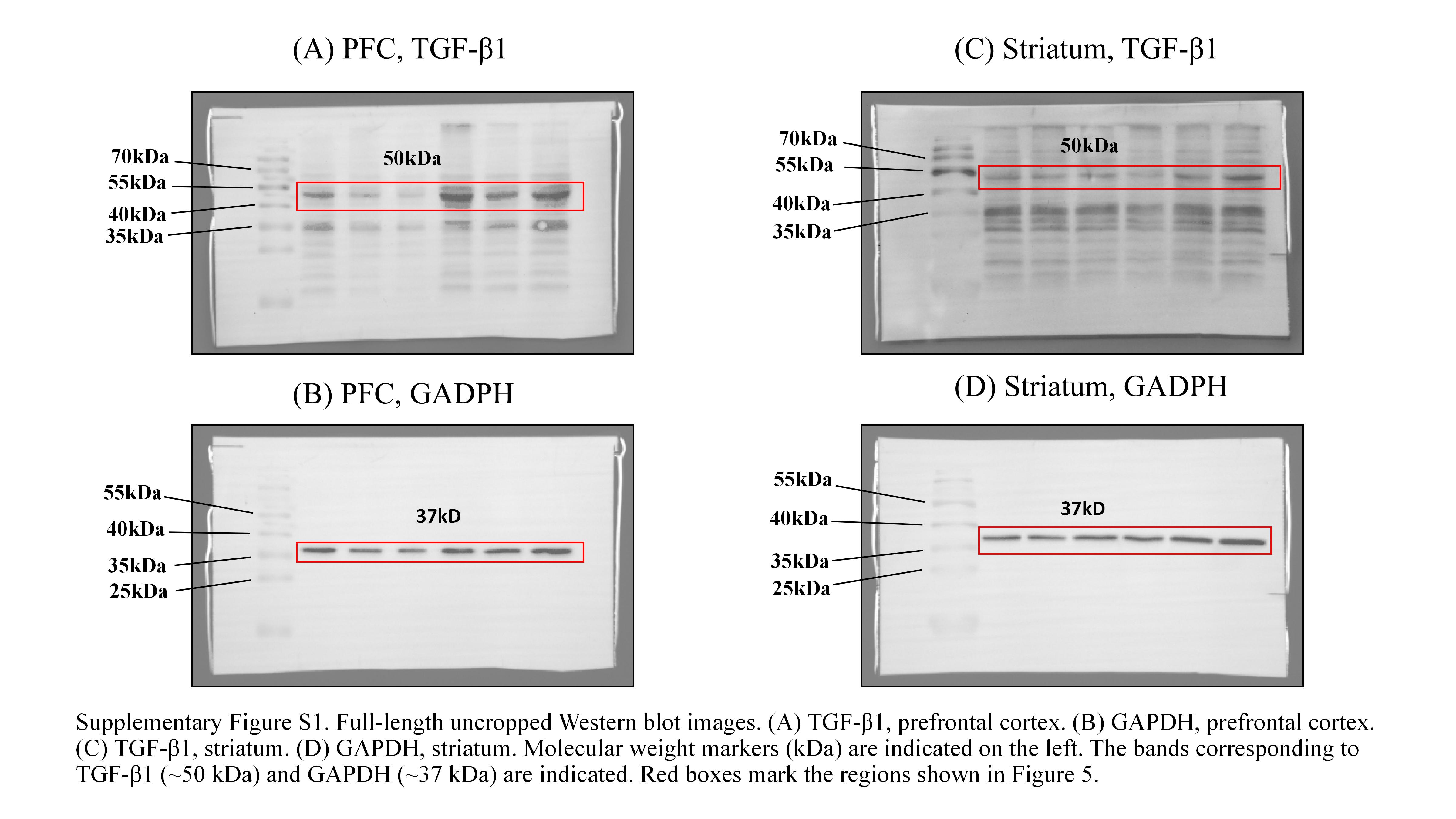

Supplement: Supplementary file 1 [file Supplementaryfile1.zip › Supplementary Figure 1.JPEG]
